# Locus specific engineering of tandem DNA repeats in the genome of *Saccharomyces cerevisiae* using CRISPR/Cas9 and overlapping oligonucleotides

**DOI:** 10.1038/s41598-018-25508-3

**Published:** 2018-05-08

**Authors:** Astrid Lancrey, Alexandra Joubert, Jean-Baptiste Boulé

**Affiliations:** 1 Genome Structure and Instability laboratory, CNRS UMR 7196, Inserm U1154, National Museum of Natural History, Life Adaptations department, 75005 Paris, France; 2 Sorbonne Universités, Paris, France

## Abstract

DNA repeats constitute a large part of genomes of multicellular eucaryotes. For a longtime considered as junk DNA, their role in genome organization and tuning of gene expression is being increasingly documented. Synthetic biology has so far largely ignored DNA repeats as regulatory elements to manipulate functions in engineered genomes. The yeast *Saccharomyces cerevisiae* has been a workhorse of synthetic biology, owing to its genetic tractability. Here we demonstrate the ability to synthetize, in a simple manner, tandem DNA repeats of various size by Cas9-assisted oligonucleotide *in vivo* assembly in this organism. We show that long tandem DNA repeats of several kilobases can be assembled in one step for different monomer size and G/C content. The combinatorial nature of the approach allows exploring a wide variety of design for building synthetic tandem repeated DNA directly at a given locus in the *Saccharomyces cerevisiae* genome. This approach provides a simple way to incorporate tandem DNA repeat in synthetic genome designs to implement regulatory functions.

## Introduction

Tandem DNA repeats constitute a large fraction of eukaryotic genomes^1^. This fraction of eukaryotic genomes has long interested a large research community, with questions ranging from their evolutionary origin and function^2^, their epigenetic effect on gene expression^3^ to their role in three dimensional genome structure^4^. Interest in their evolutionary dynamics also stems from their role as markers for genetic footprinting^5^ and in human diseases, like Huntington disease, myotonic dystrophy type 1 and several neurodegenerative diseases^6,7^. Yeast has long been a model of choice to study the biology of tandem DNA repeats. Large cloned arrays of human tandem DNA repeats inserted into the yeast genome^8–10^ or carried by a plasmid^11^ are stable across a wide array of size in a wild type background, allowing for identification of genetic pathways associated to repeat instability. Analyses of yeast mutant strains carrying tandem DNA repeats have allowed a better understanding of the roles of replication, DNA repair, recombination, transcription or DNA structures in genetic stability of trinucleotide repeats^12^ or human G-rich minisatellites^9,10^.

*S*. *cerevisiae* genome contains natural tandem DNA repeats that have been studied for their potential functional role and capacity to evolve under phenotypic selection^13–15^. A classical example is the FLO genes locus, in which copy number of FLO genes influence the flocculation phenotype and cell adherence to surface^15,16^. Another well-studied example is the copy number of rDNA genes, which can vary greatly between natural strains. Copy number variation within the rDNA locus has served as a model to understand concerted evolution of repeated DNA sequences or the role of replication stress in influencing copy number^13,17^. A measurable phenotypic effect of tandem repeat copy number variation (CNV) has also been shown for short nucleotide repeats located inside yeast promoters^18^. These studies highlight the potential of *S*. *cerevisiae* for testing phenotypic consequences of CNV of given tandem DNA repeats.

Experimental approaches to insert synthetic tandem DNA repeats in the yeast genome have been devised in the past. To generate the repeats, the main experimental approaches rely on an *in vitro* step, either by enzymatic ligation of monomers^19^, by polymerase chain reaction (PCR)^9^ or by rolling circle amplification (RCA)^11^. Synthesis by ligation allows controlling the number of repetition to be assembled, whereas PCR and RCA allow generating larger repeats. In the case of PCR, sequence heterogeneity of the repeat monomer is often large, owing to PCR frequent mispriming, whereas with RCA long sizes of faithfully replicated tandem repeats can be achieved. However, this technique is not well suited to engineer polymorphic repeats. To circumvent some of the limitations offered by the existing methods, we reasoned that a more versatile approach to generate synthetic DNA repeated arrays inside the yeast genome should be advantageous for many synthetic biology projects. Thanks to the efficiency of homologous recombination in *S*. *cerevisiae*, many techniques allowing direct DNA assembly in yeast using donor DNA from plasmids, PCR products or oligonucleotides have been proposed in the past^20–22^. These also include recent technologies of genome editing relying on CRISPR/Cas9 enzymes to selectively drive assembly of donor DNA at an induced double stranded break. CRISPR/Cas9 allows easier scar-less genome editing, improves editing efficiency, and eases multiplex genome editing when compared to older techniques^23–28^.

In this report, we show that a simple experimental approach using CRISPR/Cas9 to assist insertion of partially overlapping oligonucleotides allows to generate in one experimental step a diverse library of tandem DNA repeat arrays, ranging typically from 1 to about 100 repeats in size. We show that the efficiency depends on the size of the monomer and to a lesser extent to the G/C content of the repeats. In particular, the approach was successful for building repeated arrays from monomers ranging in size from 46 to 165 base pair (bp). The approach should be useful for synthetic chromatin or promoter engineering, or functional genomics studies.

## Results

### Experimental Design

**Figure 1:**
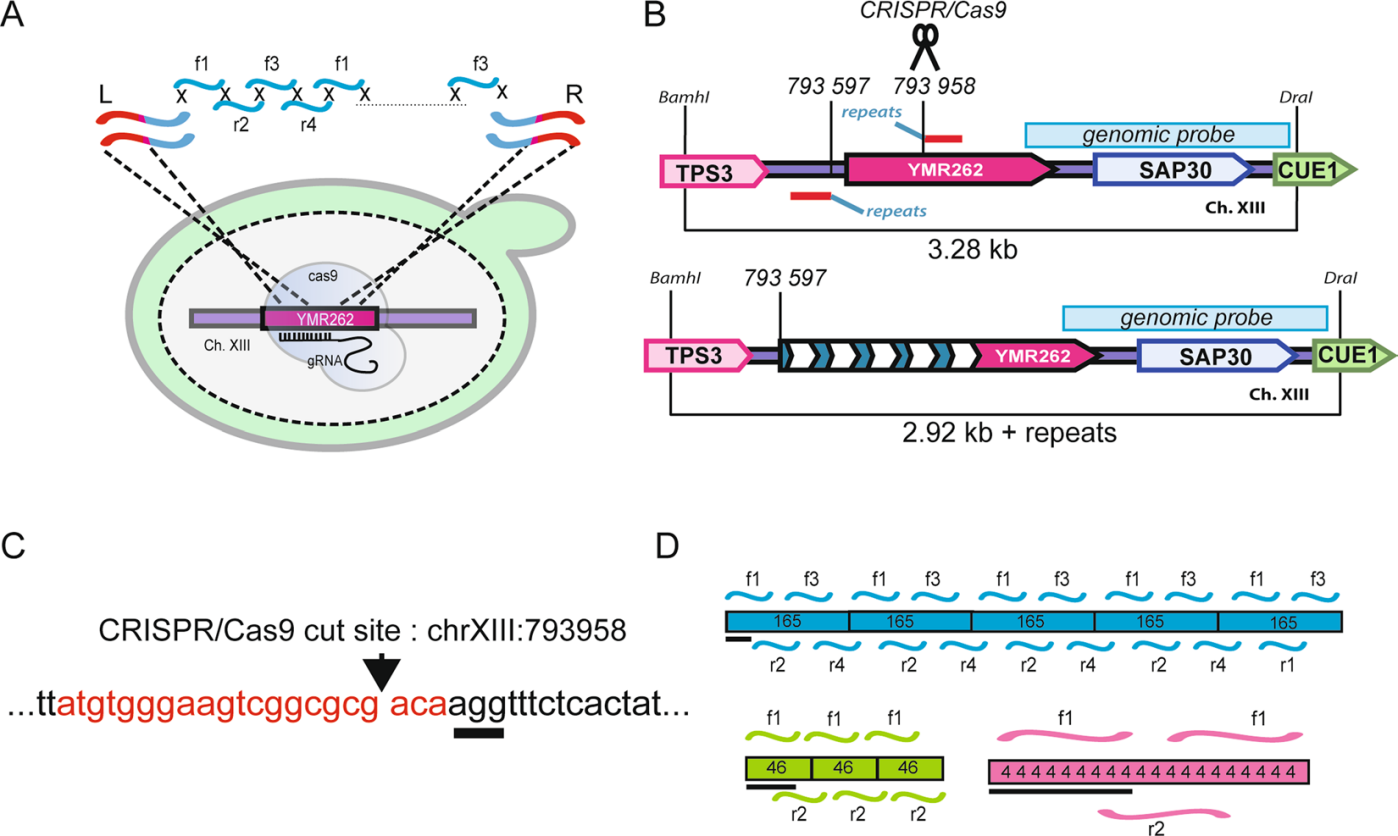
*In vivo* assembly of tandem DNA repeats. **(A)** Schematic representation of the CRISPR/Cas9-assisted integration of overlapping oligonucleotides into a specific locus in the yeast genome. As an example, the method is represented for the assembly of arrays of 165 bp-long DNA repeats in the YMR262 gene. L and R design respectively the left and right donor DNA PCRs. **(B)** Map of the YMR262 locus. Coding sequences of TPS3, SAP30 and CUE1 neighboring genes included in the BamhI-DraI fragment are indicated. The localization of the genomic probe used for Southern blotting is shown by a light blue rectangle. The localization of the genomic junction PCRs are indicated by thick red and blue lines. Coordinates of junctions between genomic and repeat DNA prior repeat insertion are indicated. The position at bp 793858 corresponds also to the CRISPR/Cas9 cut site. The respective sizes of the wild-type (top panel) and edited (bottom panel) BamhI-DraI fragments are indicated. **(C)** Sequence surrounding the cutting site. The 20 nt guide sequence is indicated in red, the PAM motif is underlined, and the cutting site is indicated by an arrowhead. **(D)** Design of the repeats: four 60 nt oligonucleotides (f1, r2, f3, r4) overlapping on 19–20 nucleotides are used to synthetize one 165 repeat unit. The 20 last nucleotides on f4 overlapp with the 20 first nucleotides on f1 so that several repeats can self assemble to form tandem repeat DNA arrays. Two overlapping oligonucleotides were used to construct arrays of 46 bp and 4 bp repeats. the black bar underneath each design shows the extent of overlap between the assembled repeat and the donor DNA used to promote oligonucleotide insertion and repair at the YMR262 locus. Oligonucleotides are shown at scale relative to the repeats.

Tandem DNA repeats can be theoretically assembled from partially complementary oligonucleotides (Fig. 1). To assemble synthetic tandem DNA repeats directly at a specific locus in the yeast genome, we reasoned that we could assemble partially overlapping oligonucleotides at the site of a DNA double strand break generated *in vivo* by a CRISPR/Cas9 complex. We chose to target assembly of synthetic repeats into the non essential YMR262 gene of chromosome XIII, which does not contain natural repeats (Fig. 1B). To that goal, we first expressed constitutively from a centromeric plasmid an engineered version of Cas9 that previously promoted efficient genome edition in yeast^24,29^ (see Methods). In a second step this Cas9 expressing strain was co-transformed with a 2*μ* plasmid constitutively transcribing a guide RNA targeting codon 79 in the YMR262 gene as the Cas9 cutting site, partially complementary oligonucleotides promoting repeat assembly, and two donor DNA fragments containing complementary regions to both genomic sequence surrounding the double DNA strand break and to the assembled repeats (Fig. 1A). The exact coordinates of the cutting site in the sacCer3 version of the S288c reference genome assembly is chrXIII:793958 (Fig. 1C). The two PCR-generated donor DNAs were homologous to 35–39 bp on the DNA repeat sequence to be assembled, and homologous to respectively 246 bp and 100 bp on genomic DNA. The left PCR was designed so that 361 bp of genomic DNA including the YMR262 promoter are lost upon repair, thus preventing influence of transcriptional activity on assembled repeats (*see* Methods, Fig. 1A and Supplementary Table s4). To test the generality of the method for generating repeats of various size and nucleotide composition, we tested assembly of G/C poor (25% G/C), G/C neutral (50% G/C) and GC rich (75% G/C) synthetic repeats of random sequence. For each G/C content, we tested three monomer length of 4, 46 and 165 bp, giving 9 designs in total. The nine expected monomer sequences are given in Supplementary Table s1. Before building the repeats, we verified using a blast query against the S288c reference genome (sacCer3 version) that the six engineered 46 and 165 bp repeats were not already present in the *S*. *cerevisiae* genome, since this could have been a source of recombination artefacts. Monomers of this size are often found in nature in promoters, genes and non-coding satellite DNA^1^. The design of overlapping oligonucleotides used to assemble tandem DNA repeats of random sequence and of three different monomer length is given in Fig. 1D. Repeat monomer sequences were broken down in 40 to 60 mer oligonucleotides overlapping on 18–20 nucleotides, the last oligonucleotide overlapping with the first one, so that a repeated assembly can take place. The number of oligonucleotides used depended on the size of the monomer, two oligonucleotides being sufficient to assemble 4 bp and 46 bp repeats and 4 oligonucleotides to assemble 165 bp repeats. In the case of the GC neutral 4 bp repeat, only one oligonucleotide was necessary in the design, since the ATGC 4 bp repeat is self complementary (Supplementary Table s4).

### Characterisation of the *in vivo* assembled repeats

**Figure 2:**
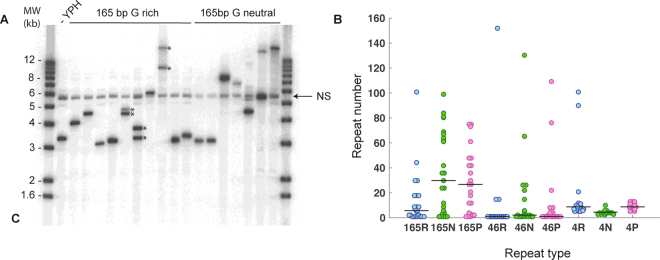
Analysis of tandem DNA repeats assembled at the YMR262 locus. **(A)** Southern blot analysis of genomic DNA from wild type YPH499 and edited strains containing DNA repeats of 165 bp G/C rich or G/C neutral repeats. Molecular weight (MW) is indicated in kb. In some clones, secondary rearrangements can be observed, leading to two repeated arrays of different sizes (indicated by *). NS denotes on non specific hybridization present in all clones including with YPH DNA lane 1). **(B)** Beeswarm plots showing the size distribution of tandem repeats assessed by Southern blotting and Sanger sequencing for each type of repeat. The horizontal black line in each plots marks the median size of the arrays. Sequences of the analyzed clones are available in Supplementary Figs s1–s9.

After transformation, cells were directly plated on selective media to transiently maintain plasmids expressing Cas9 and the guide RNA. In these conditions, only yeasts having repaired the double strand break by modification of the cutting site should survive^24^ (Fig. 1A). In our hands, each transformation events of 10^6^ yeast cells yielded more than two hundred surviving clones. For comparison, transformation of the guide RNA only yields less than 10 colonies in average (Supplementary Fig. s10). From each transformation experiment, 10 clones were picked and analyzed by southern blotting. Results of two independent transformations are shown in Fig. 2. As exemplified in Fig. 2A, each transformant analyzed carried a modified YMR262 allele. Sizes of inserted DNA at the YMR262 locus varied widely depending on the type of repeat assembled. Assembled repeats up to 1 kb long were PCR amplified to allow precise size measurement after Sanger sequencing. For longer repeats, arrays were sized directly from analysis of the southern blot. Assembly of 165 bp repeats using a design of four partially complementary oligonucleotides yielded the largest size diversity of recombinant insertions, with inserts ranging from one repeat to arrays larger than 15 kb, corresponding to an assembly of around a hundred monomers. The assembly of a 46 bp repeats using a two oligonucleotides design led mainly to assembly of a single repeat. This is not completely unexpected, given that the two donor DNAs are partially overlapping over about 24–26 bp by design, allowing the insertion of single repeats. However, in each case (G/C poor, neutral or rich), at least a long insertion was recovered, indicating that this approach allows synthesis of long repeats, yet at a lower efficiency than the four oligonucleotides design. The assembly of 4 bp repeats led predominantly to short assembled repeats (5–10 repeats). As in the case of 46 bp repeats, assembly of 5–10 repeats can be explained by direct recombination of overlapping sequences within donor DNA without insertion of overlapping oligonucleotides. However, long insertion of up to 100 repeats were recovered from the assembly of 4 bp GC rich monomers, demonstrating that synthesis of large repeated arrays by oligonucleotide assembly is feasible in this context.

### Fidelity of *in vivo* repeat assembly

**Figure 3:**
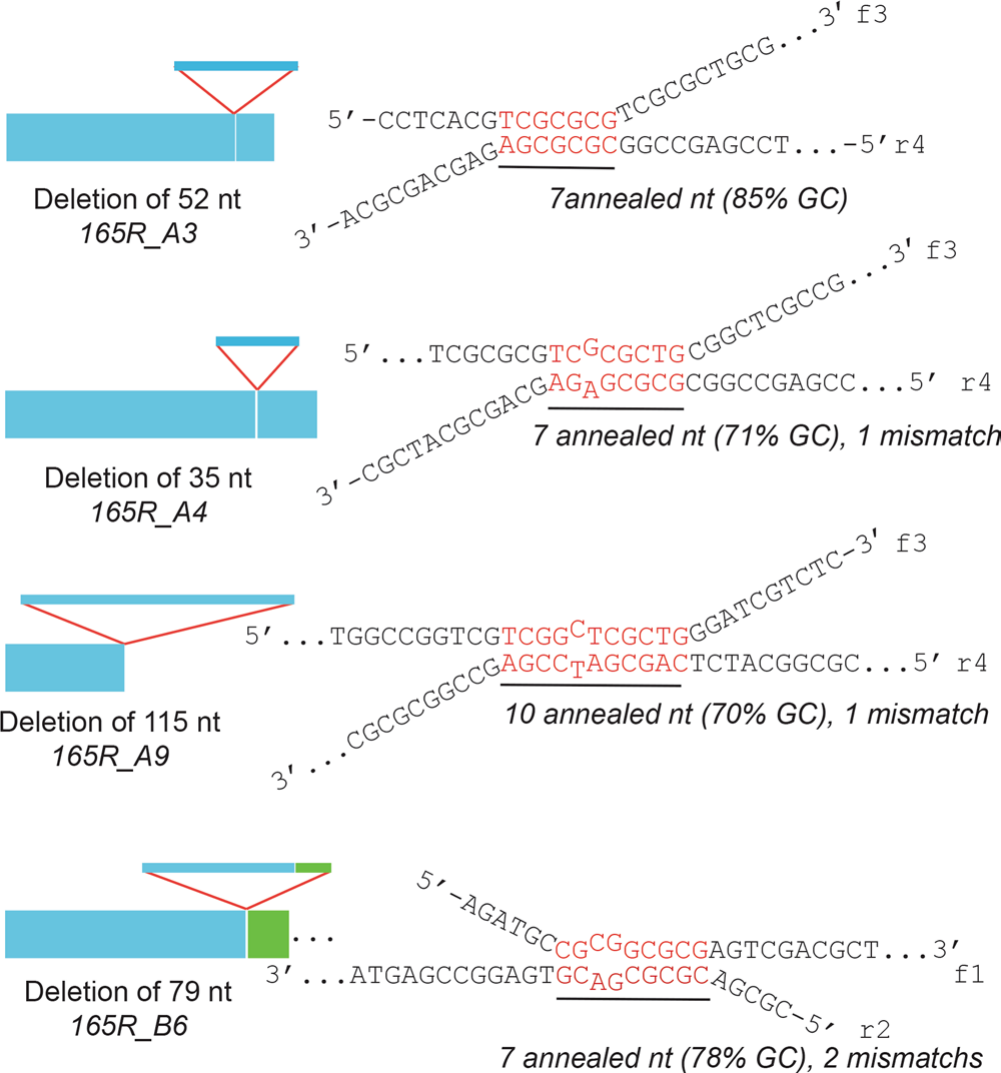
Origin of large deletions in G/C rich 165 bp DNA arrays. Left panel. Exemple analysis of 4 edited clones showing large deletions in the intended monomer sequence. A monomer is depicted by a blue rectangle, indicating the position and relative size of each deletion observed. Edited clone number is indicated and the sequence can be found in Supplementary Fig. s9. (Right panel). Proposed oligonucleotide annealing explaining observed monomer sequence variability in G/C rich 165 bp tandem DNA arrays.

Repeats longer than 1 kb were out of reach for direct PCR amplification and Sanger sequencing. To check that the insertions were true repeats and verify the fidelity of the assembly process, we PCR amplified repeats shorter than 1 kb including genomic junctions. Amplified repeats were analyzed by Sanger sequencing for the 9 different designs. Sequences of isolated clones were scored for single nucleotide polymorphisms, insertions and deletions. Results are summarized in Table 1 and Supplementary Figs s1–s9. Out of a total of 262 genomic DNA-repeat junctions sequenced, we found only four substitutions, six indels and one insertion of five nucleotides, indicating that the fidelity of the insertion process is very high. Regarding the repeats, the accuracy and fidelity of tandem repeat assembly varied slightly depending on the G/C content. The least accurate assembly was observed for the G/C rich 165 bp repeats. G/C poor repeat tended to have increased nucleotide substitutions and indel. These errors can be explained by misalignment of two oligonucleotides during annealing and/or recombination insertion. For the G/C rich 165 bp repeat, the main errors were large deletions of 5, 53, 64 and 115 bp, present in 5 out of 16 sequenced clones. For this particular repeat, we wondered if this high error rate was also due to misalignement of the repeat-coding oligonucleotides. As shown in Fig. 3, the deletions observed can be straightforwardly explained by spurious annealing of two oligonucleotides over a short G/C rich stretch of 7–10 complementary bases. This result suggests that short G/C rich 10 nucleotides complementary stretches are sufficient to drive oligonucleotide assembly, adding versatility into oligonucleotide designs that can be implemented to drive repeat assembly with this approach.

**Table 1:** Fidelity of the assembly process.

Repeats	4P	4N	4R	46P	46N	46R	165P	165N	165R
#clones	19	15	20	17	16	16	11	15	16
#sequenced nt	728	280	1072	2252	1445	734	4279	8033	2802
substitutions	0	1	0	0	0	1	3	19	2
indels	0	0	0	10	1	0	3	3	4
deletions >3 *nt*	0	0	0	1	1	1	1	1	7
insertions >3 *nt*	0	0	0	0	0	0	0	0	1
Junction (50 bp up and downstream)									
#clones	19	13	20	16	16	16	10	11	15
substitutions	0	0	0	0	1	0	3	0	0
indels	0	0	2	0	0	0	4	0	0
deletions >3 *nt*	0	0	0	0	0	0	0	0	0
insertions >3 *nt*	0	0	0	0	0	1	0	0	0

Number of substitutions, indels, insertions and deletions measured from sequencing 145 clones covering the 9 different repeat designs tested of 4, 46 and 165 bp. P stands for GC Poor, N for G/C Neutral and R for G/C rich. Sequences used to create this table are given in Supplementary Figs s1–s9.

## Discussion

The goal of this study was to provide a simple experimental way to build tandem DNA repeats of various sizes in the genome of *S*. *cerevisiae*. We showed that it can be achieved in one experimental step by using CRISPR/Cas9 and partially overlapping oligonucleotides. The use of CRISPR/Cas9 greatly simplifies genome editing even in genetically tractable organisms like *Saccharomyces cerevisiae*. Here we show that we can engineer, using CRISPR/Cas9, a combinatorial library of different size of DNA repeat arrays (typically from one to a hundred repeats) in a single step experiment, for different monomer size and GC content. Interestingly, we observed that short GC rich stretches of 7–10 nucleotides are sufficient for oligonucleotide annealing. In our experience, short GC rich stretches homologous between oligonucleotides introduced sequence variability within the assembled repeats. This suggests that overlapping sequences between oligonucleotides used to drive repeat assembly could be shortened from 20 to stretches of 10 G/C nucleotides. The approach was not very efficient for building arrays of tetranucleotide repeats that were diverse in size. Therefore, alternative oligonucleotide designs could be explored for microsatellite design, for example by reducing the size of the region complementary to the assembled repeats within the donor DNA, to minimize direct repair by donor DNA without oligonucleotide insertion.

In this study, we only sequenced shorter repeats amenable to PCR amplification and Sanger sequencing, but a different strategy would have to be implemented for the sequencing of longer repeats. Long read sequencing technologies like nanopore sequencing or SMRT sequencing can today circumvent issues posed by sequencing of long tandem DNA repeats^30–32^.

**Figure 4:**
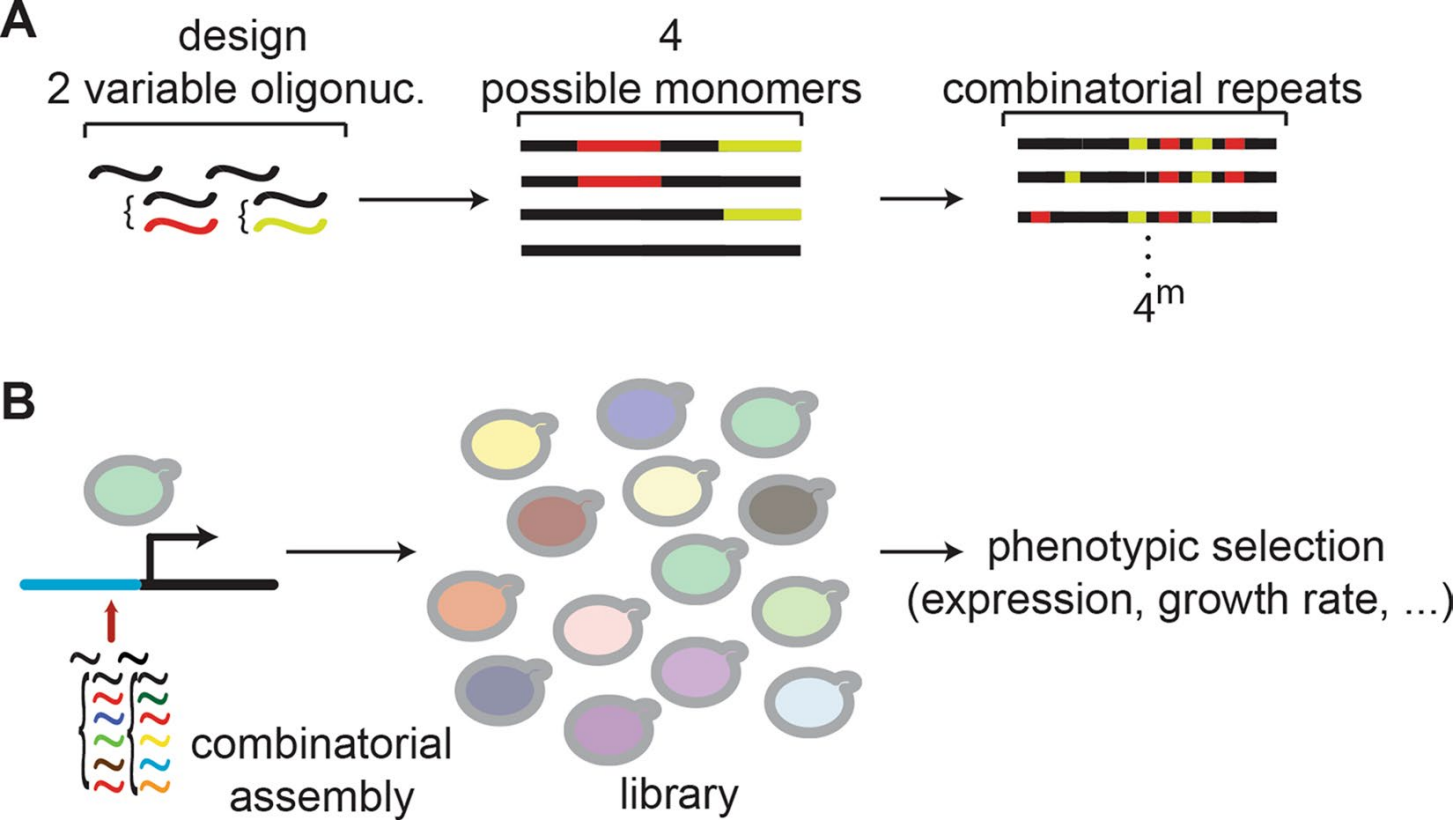
Engineering of a combinatorial library of tandem DNA repeat arrays. **(A)** Example is given for a four oligonucleotide design with two variable oligonucleotides. m being the number of repeat in a given array, there are 4^*m*^ possible arrays of length m. **(B)** Example strategy to design a combinatorial library of promoter sequences, using a collection of oligonucleotides with diverse transcription factor binding sites. Upon transformation, the library can be selected based on gene expression, fitness, or other selectable phenotype.

In conclusion, beyond building homologous tandem DNA repeats, the approach should allow engineering combinatorial libraries of heterogenous repeats that can be selected through an appropriate phenotypic screening of the recombinant cells (Fig. 4). Drawing examples from nature, tandem DNA repeats could be used for example to build spacer DNA between genetic elements^33^ or for bottom-up engineering of regulatory regions, like promoters and enhancers^34,35^. Indeed, the approach presented here provides an alternative to combinatorial design of promoters^34^, *e*.*g*. with the introduction of transcription factor binding sequences in non overlapping oligonucleotide regions. Since repeats are assembled statistically from the oligonucleotide mix provided during transformation, providing alternative oligonucleotide sequences located in non overlapping regions of the repeat should be straightforward to increase the diversity of the library assembled into the repeated array. For example, the oligonucleotide designs proposed here for 46 bp and 165 bp monomers allow respectively 9 and twice 22 non overlapping nucleotides where diverse binding sites can be incorporated. Noteworthy, this approach should also be an interesting addition to the techniques developed by the Sc2.0 project (www.syntheticyeast.org) for the synthesis of a yeast synthetic genome, allowing introduction of repeated sequences in the synthetically assembled building blocks. Our technique therefore prevents the need to design repeats in the synthetics “chunks”^36^, as they can be assembled directly *in vivo* following sequence replacement. The only limitation of our technique is that it does not allow to predetermine the exact number of repeats added. However, given the large number of repeats that can be added by this approach, it might still be cost effective to select from a large library of engineered repeats as opposed to fully construct large repeats *in vitro*. Finally, the approach would be easily testable in other systems than *S*. *cerevisiae*, depending on the ability of the host system to favor homologous recombination over non-homologous end joining to repair DNA double strand breaks.

## Methods

### Strains, Plasmids, Media and Primers

The *Saccharomyces cerevisiae* strain YPH499 (ATCC # 204679) was used as a parental strain throughout the study. YPH499 derived strains and plasmids relevant to this study are listed in Supplementary Tables s2 and s3, respectively. Strains were grown either in YPA-2% glucose medium or in Complete Synthetic media (CSM) containing 2% glucose and lacking appropriate amino acids supplements in order to maintain plasmid-borne auxotrophic markers. All media reagents were purchased from Formedium, UK. Cells were grown at 30 °C in a New Brunswick orbital incubator shaker at 200 rpm. All primers used in this study were synthetized by Eurogentec and are listed in Supplementary Table S4.

### Oligonucleotide design for *in vivo* genomic repeat assembly

DNA repeats were assembled *in vivo* from overlapping oligonucleotides (Supplementary Table s4). Oligonucleotides (45–60 nucleotides long) were designed to allow combinatorial assembly by partial overlap of 19–20 nucleotides. For each repeat length (4, 46 or 165 bp), oligonucleotide combinations were designed in order to create G/C-poor (25%G/C), GC-neutral (50%G/C) or G/C-rich (75% G/C) tandem DNA repeats of arbitrary sequence.

### Synthesis of the guide RNA expression and Cas9 expression plasmids

The CRISPOR tool (http://crispor.tefor.net/)^37^ was used to select guide RNA sequences for targeting the YMR262 locus without any off-target (Supplementary Table s4). To create the guide RNA expression plasmid, we used p426-SNR52p-gRNA.CAN1.Y-SUP4t (Addgene reference #43803^24^) and replaced the 20 bp gRNA CAN1 target sequence by the YMR262 target sequence. First, the backbone of vector p426-SNR52p-gRNA.CAN1.Y-SUP4t was amplified by PCR with primers AL-O-44 and AL-O-45 using the Phusion HF DNA polymerase (New England Biolabs). The backbone was digested with ClaI and KpnI to create compatible ends for cloning. A claI/KpnI fragment was synthetized by PCR using primers AL-O-46 and AL-O-47 and p426-SNR52p-gRNA.CAN1.Y-SUP4t (Addgene # 42803) as a template. Upon cloning between ClaI and KpnI sites, the resulting plasmid (pAL31) contains the 20 bp gRNA genomic target sequence under control of the SNR52 promoter, followed by the tracrRNA sequence and the SUP4 terminator. To construct a Cas9 expression plasmid containing a HIS selection marker, p414-TEF1p-Cas9-CYC1t (Addgene #43802) and pRS413^38^ were cut with enzymes AhdI and DraIII (New England Biolabs). The backbone of pRS413 containing the CEN/ARS sequence and both AmpR and HIS3 selection markers was gel purified and ligated to the fragment of p414-TEF1p-Cas9-CYC1t containing the human optimized Cas9 gene under the TEF1p promoter (pAL30). The correct sequence of the CAS9 gene was verified by Sanger sequencing.

### *In vivo* repeat assembly

Donor DNAs corresponding to left and right genomic/repeats junctions were synthesized by PCR with the Phusion HF DNA polymerase (New England Bioloabs) using primer couples AL-O-24/25–33 and AL-O-43/34–42, with YPH499 genomic DNA as a template. The left junction was designed so that the YMR262 promoter and the beginning of the gene are lost upon recombinational insertion (Fig. 1B). The YPH499-Cas9 strain was created by transforming plasmid pAL30 into YPH499 by the LiAC protocol, to form the ALY0 strain^39^. *In vivo* synthesis of tandem DNA repeats at the YMR262 locus was achieved by transformation of yeast spheroplasts prepared according to the procedure established by the Larionov lab^40^ with little modifications. Briefly, the Cas9-expressing ALY0 strain was grown in SCD-His to an OD_600_ of 0.6–0.8. 10^7^ spherloplasts were transformed with 1 *μ*g of plasmid pAL31, 100 pmol of each repeat-forming oligonucleotide (AL-O-01 to 23, Supplementary Table s4), and 10 pmol of both donor DNAs. After transformation cells were plated on SCD-His-Ura and incubated 48 to 72 hours at 30 °C to allow growth of surviving clones containing repeat insertions at the site of CRISPR/Cas9 cutting. Growing clones were first reisolated on SCD-His-URA before screening.

### Screening of edited clones by Southern blotting and Sanger sequencing

Screening was done by purification of genomic DNA from yeast transformants followed by Southern blotting. Genomic DNAs from twenty clones of each repeat design were digested with BamHI and DraI, which cut at each side of the insertion locus (Fig. 1B). For the Southern blots, we used a 1 kb long P^32^-radio-labeled probe synthetized by PCR with primers AL-O-50 and 51 (Fig. 1B and Supplementary Table s4) using YPH499 genomic DNA as a template. This probe reveals a band migrating at 3,28 kb from BamHI/DraI-digested YPH499 genomic DNA. If a repeat has been assembled *in vivo* upon transformation, the size of the band revealed by this probe should be 2.92 kb plus the size of the integrated repeated array. Sanger sequencing of the repeat, including around 100 bp of surrounding genomic DNA, was performed on PCR products using primers listed in Supplementary Table s4 and the Phusion DNA polymerase (New England Biolabs), using genomic DNA from edited clones as a template. Whenever PCR products of multiple sizes were generated during the PCR reaction, the main band was purified before Sanger sequencing. This did not happen systematically however, and most of the PCR reactions yielded products of unique size in our hands. Sanger sequencing was performed by GATC biotech. Sequences are available in Supplementary Figs s1–s9.

## Electronic supplementary material

Supplementary Material

## References

[1] Richard G-F, Kerrest A, Dujon B (2008). Comparative genomics and molecular dynamics of DNA repeats in eukaryotes. Microbiol. Mol. Biol. Rev..

[2] Shapiro JA, von Sternberg R (2005). Why repetitive DNA is essential to genome function. Biol. Rev. Camb. Philos. Soc..

[3] Pezer, Z., Brajkovic, J., Feliciello, I. & Ugarkovc, D. Satellite DNA-Mediated Effects on Genome Regulation in *Repetitive DNA. Genome dyn.* (ed. Garrido-Ramos, M.A.) vol. 7, 153–169 (Karger, 2012). 10.1159/000337116 22759818

[4] Cournac A, Koszul R, Mozziconacci J (2016). The 3D folding of metazoan genomes correlates with the association of similar repetitive elements. Nucleic Acids Res.

[5] Gyapay G (1994). The 1993–94 Genethon human genetic linkage map. Nat. Genet..

[6] Kim JC, Mirkin SM (2013). The balancing act of DNA repeat expansions. Curr. Op. Gen. Dev.

[7] Schmidt MH, Pearson CE (2016). Disease-associated repeat instability and mismatch repair. DNA Repair.

[8] Freudenreich CH, Kantrow SM, Zakian VA (1998). Expansion and length-dependent fragility of CTG repeats in yeast. Science.

[9] Ribeyre C (2009). The yeast Pif1 helicase prevents genomic instability caused by G-quadruplex-forming CEB1 sequences in vivo. PLoS Genet..

[10] Lopes J (2011). G-quadruplex-induced instability during leading-strand replication. EMBO j.

[11] Ebersole T (2005). Rapid generation of long synthetic tandem repeats and its application for analysis in human artificial chromosome formation. Nucleic Acids Res..

[12] Williams GM, Surtees JA (2018). Measuring dynamic behavior of trinucleotide repeat tracts in vivo in Saccharomyces cerevisiae. Methods Mol. Biol.

[13] Ganley AR, Kobayashi T (2007). Highly efficient concerted evolution in the ribosomal DNA repeats: Total rDNA repeat variation revealed by whole-genome shotgun sequence data. Genome Res..

[14] Rolland T, Dujon B, Richard G-F (2010). Dynamic evolution of megasatellites in yeasts. Nucleic Acids Res.

[15] Verstrepen KJ, Jansen A, Lewitter F, Fink GR (2005). Intragenic tandem repeats generate functional variability. Nat. Genet..

[16] Rando OJ, Verstrepen KJ (2007). Timescales of genetic and epigenetic inheritance. Cell.

[17] Salim D (2017). DNA replication stress restricts ribosomal DNA copy number. PLoS Genet..

[18] Vinces MD, Legendre M, Caldara M, Hagihara M, Verstrepen KJ (2009). Unstable tandem repeats in promoters confer transcriptional evolvability. Science.

[19] Scior A, Preissler S, Koch M, Deuerling E (2011). Directed PCR-free engineering of highly repetitive DNA sequences. BMC Biotechnol..

[20] DiCarlo JE (2013). Yeast Oligo-Mediated Genome Engineering (YOGE). ACS Synth. Biol..

[21] Gibson DG (2011). Gene and genome construction in yeast. Curr. Protoc. Mol. Biol..

[22] Stuckey S, Mukherjee K, Storici F (2011). In vivo site-specific mutagenesis and gene collage using the delitto perfetto system in yeast Saccharomyces cerevisiae. Methods Mol. Biol.

[23] Bao Z (2015). Homology-integrated CRISPR-Cas (HI-CRISPR) system for one-step multigene disruption in Saccharomyces cerevisiae. ACS Synth. Biol..

[24] DiCarlo JE (2013). Genome engineering in Saccharomyces cerevisiae using CRISPR-Cas systems. Nucleic Acids Res.

[25] Laughery MF (2015). New vectors for simple and streamlined CRISPR-Cas9 genome editing in Saccharomyces cerevisiae. Yeast.

[26] Mans R (2015). CRISPR/Cas9: a molecular Swiss army knife for simultaneous introduction of multiple genetic modifications in Saccharomyces cerevisiae. FEMS Yeast Res..

[27] Ronda C (2015). CrEdit: CRISPR mediated multi-loci gene integration in Saccharomyces cerevisiae. Microb. Cell Fact..

[28] Si T (2017). Automated multiplex genome-scale engineering in yeast. Nat. Commun..

[29] Mali P (2013). RNA-guided human genome engineering via Cas9. Science.

[30] Mitsuhashi S (2017). Nanopore-based single molecule sequencing of the D4Z4 array responsible for facioscapulohumeral muscular dystrophy. Sci. Reports.

[31] McGinty RJ (2017). Nanopore sequencing of complex genomic rearrangements in yeast reveals mechanisms of repeat-mediated double-strand break repair. Genome Res..

[32] Ogeh D, Badge R (2017). A pipeline for local assembly of minisatellite alleles from single-molecule sequencing data. Bioinformatics.

[33] Dobi KC, Winston F (2007). Analysis of transcriptional activation at a distance in Saccharomyces cerevisiae. Mol. Cell. Biol..

[34] Dossani ZY (2018). A combinatorial approach to synthetic transcription factor-promoter combinations for yeast strain engineering. Yeast.

[35] Amit R, Garcia H, Phillips R, Fraser S (2011). Building enhancers from the ground up: a synthetic biology approach. Cell.

[36] Shen Y (2017). Deep functional analysis of synII, a 770-kilobase synthetic yeast chromosome. Science.

[37] Haeussler M (2016). Evaluation of off-target and on-target scoring algorithms and integration into the guide RNA selection tool CRISPOR. Genome Biol..

[38] Sikorski RS, Hieter P (1989). A system of shuttle vectors and yeast host strains designed for efficient manipulation of DNA in Saccharomyces cerevisiae. Genetics.

[39] Hill J, Donald KA, Griffiths DE, Donald G (1991). DMSO-enhanced whole cell yeast transformation. Nucleic Acids Res.

[40] Kouprina N, Noskov VN, Larionov V (2006). Selective isolation of large chromosomal regions by transformation-associated recombination cloning of mammalian genomes. Methods Mol. Biol..

