# Supplementary material for: Locus specific engineering of tandem DNA repeats in the genome of Saccharomyces cerevisiae using CRISPR/Cas9 and overlapping oligonucleotides
Source: Sci Rep. 2018 May 8;8:7127. doi: 10.1038/s41598-018-25508-3 (PMC5940919; doi:10.1038/s41598-018-25508-3)
Supplement: Supplementary file 1 — Supplementary Material [file 41598_2018_25508_MOESM1_ESM.pdf]

Locus specific engineering of tandem DNA repeats in the genome of *Saccharomyces cerevisiae* using CRISPR/Cas9 and overlapping oligonucleotides

Astrid Lancrey, Alexandra Joubert and Jean-Baptiste Boulé

Page 2 Supplementary Figures s1-s10

Page 12 Supplementary information, containing supplementary Tables s1-s4 with relevant information on strains, plasmid and oligonucleotides used in this study.

A.

A3,A6,B2,B4	ATGA ATGA ATGA ATGA ATGA ATGA ATGA ATGA ATGA
A4,A9	ATGA ATGA ATGA ATGA ATGA ATGA ATGA ATGA
A5	ATGA ATGA ATGA ATGA ATGA ATGA
A1,A2,A8	ATGA ATGA ATGA ATGA ATGA ATGA ATGA ATGA ATGA ATGA ATGA ATGA
A10,B7	ATGA ATGA ATGA ATGA ATGA ATGA ATGA ATGA ATGA ATGA ATGA
B1,B6,B10	ATGA ATGA ATGA ATGA ATGA ATGA ATGA ATGA ATGA ATGA ATGA ATGA ATGA
B3,B5	ATGA ATGA ATGA ATGA ATGA
B8	ATGA ATGA ATGA ATGA ATGA ATGA ATGA
B9	ATGA ATGA ATGA ATGA ATGA ATGA ATGA ATGA ATGA ATGA

B.

19/19	TGATGCTCCCTGGAGTTGCCGTTGCTGCGTGCTGAAGGGGATTAATTAAT	ACAAGGTTTCTCACTATCAAGATGTACTGGAGTATAAGAATGAAGAACAG

Supplementary Figure s1. Sequences of edited YMR262 loci with G/C poor 4 bp repeats. **(A)** Sequences of repeats in isolated edited clones. Clone numbers are indicated on the left. **(B)** Sequence of the surrounding junctions, 50 bp upstream and downstream of the repeat sequence. Ratio on the left indicates the number of clones of a given sequence over the total number of junctions sequenced.

A.

A1,A3,A10,B3,B8,B9	ATGC ATGC ATGC
A4	ATGC ATGC ATGC ATGC ATGC ATGC ATGC ATGC
A5,A7,A8	ATGC ATGC ATGC ATGC
A6	ATGC ATGC ATGC ATGC ATGC ATGC ATGC ATGC ATGC ATGC
B1	ATGC ATGC ATG<u>*T*</u> ATGC
B6,B7,B10	ATGC ATGC ATGC ATGC ATGC ATGC

B.

13/13 TGATGCTCCCTGGAGTTGCCGTTGCTGCGTGCTGAAGGGGATTAATTAAT ACAAGGTTTCTCACTATCAAGATGTACTGGAGTATAAGAATGAAGAACAG

Supplementary Figure s2. Sequences of edited YMR262 loci with G/C neutral 4 bp repeats. **(A)** Sequences of repeat regions in isolated edited clones. Single nucleotide polymorphisms are italicized and underlined. Clone numbers are indicated on the left. **(B)** Sequence of the surrounding junctions, 50 bp upstream and downstream of the repeat sequence. Ratio on the left indicates the number of clones of a given sequence over the total number of junctions sequenced.

A.

A1 ACGC ACGC ACGC ACGC ACGC

A2,A3,B3,B9 ACGC ACGC ACGC ACGC ACGC ACGC ACGC

A5 ACGC ACGC ACGC ACGC ACGC ACGC ACGC ACGC ACGC ACGC ACGC ACGC

A6,A7 ACGC ACGC ACGC ACGC ACGC ACGC ACGC ACGC

A8 ACGC ACGC ACGC ACGC ACGC ACGC ACGC ACGC ACGC ACGC ACGC ACGC ACGC ACGC

A4 ACGC ACGC ACGC ACGC ACGC ACGC ACGC ACGC ACGC ACGC ACGC ACGC ACGC ACGC ACGC ACGC ACGC ACGC ACGC ACGC ACGC

A9,A10 ACGC ACGC ACGC ACGC ACGC ACGC ACGC ACGC ACGC ACGC

B1,B8 ACGC ACGC ACGC ACGC ACGC ACGC

B2,B4 ACGC ACGC ACGC ACGC ACGC

B5 ACGC ACGC ACGC ACGC ACGC ACGC ACGC ACGC ACGC ACGC ACGC ACGC ACGC ACGC ACGC ACGC ACGC ACGC ACGC ACGC ACGC ACGC ACGC ACGC ACGC
ACGC ACGC ACGC ACGC ACGC ACGC ACGC ACGC ACGC ACGC ACGC ACGC ACGC ACGC ACGC ACGC ACGC ACGC ACGC ACGC ACGC ACGC ACGC ACGC ACGC
ACGC ACGC ACGC ACGC ACGC ACGC ACGC ACGC ACGC ACGC ACGC ACGC ACGC ACGC ACGC ACGC ACGC ACGC ACGC ACGC ACGC ACGC ACGC ACGC ACGC
ACGC ACGC ACGC ACGC ACGC ACGC ACGC ACGC ACGC ACGC ACGC ACGC ACGC ACGC ACGC ACGC ACGC ACGC ACGC ACGC ACGC ACGC ACGC ACGC ACGC
ACGC

B6,B7 ACGC ACGC ACGC ACGC ACGC ACGC ACGC ACGC ACGC

B10 ACGC ACGC ACGC ACGC ACGC ACGC ACGC ACGC ACGC ACGC ACGC

B.

18/20 TGATGCTCCCTGGAGTTGCCGTTGCTGCGTGCTGAAGGGGATTAATTAAT ACAAGGTTTCTCACTATCAAGATGTACTGGAGTATAAGAATGAAGAACAG

B1 TGATGCTCCCTGGAGTTGCCGTTGCTGCGTGCTGAAGGGGATTAATTAA- ACAAGGTTTCTCACTATCAAGATGTACTGGAGTATAAGAATGAAGAACAG

B2 TGATGCTCCCTGGAGTTGCCGTTGCTGC-TGCTGAAGGGGATTAATTAAT ACAAGGTTTCTCACTATCAAGATGTACTGGAGTATAAGAATGAAGAACAG

Supplementary Figure s3. Sequences of edited YMR262 loci with G/C rich 4 bp repeats. **(A)** Sequences of repeat regions in isolated edited clones. Clone numbers are indicated on the left. **(B)** Sequence of the surrounding junctions, 50 bp upstream and downstream of the repeat sequence. Ratio on the left indicates the number of clones of a given sequence over the total number of junctions sequenced. Indels are indicated in grey.

A.

```
A1,A4,A5,B1,B2,B4,B6,B7,B8,B10    ATGAGAGTAACTATATTAGATCTATTAATGACTAGATAGTTAATCT

A2                                ATGAGAGTAACTATATTAGATCTATTAATGACTAGATAGTTAATCT ATGAGAGTAACTATATTAGATCTATTAATGACTAGATAGTTAATCT
                                  ATGAGAGTAACTATATTAGATCTATTAATGACTAGATAGTTAATCT ATGAGAGTAACTATATTAGATCTATTAATGACTAGATAGTTAATCT

A6                                ATGAGAGTAACTATATTAGATCTATTAATGACTAGATAGTTAATCT ATGAGAGTAACTATATTAGATCTATTAATGACTAGATAGTTAATCT

A7                                ATGAGAGTAACTATATTAGATCTATTAATGACTAGATAGTTAATCT ATGAGAGTAACTATATTAGATCTATTAATGACTAGATAGTTAAT-T
                                  ATGAGAGTAACTATATTAGATCTATTAATGACTAGATAGTTAATCT ATGAGAGTAACTATATTAGATCTATTAATGACTAGATAGTTAATCT
                                  ATGAGAGTAACTATATTAGATCTATTAATGACTAGATAGTTAAT-T

A8                                ATGAGAGTAACTATATTAGATCTATTAATGACTAGATAGTTAAT-T ATGAGAGTAACTATATTAGATCTATTAATGACTAGATAGTTA----
                                  -TGAGAGTAACTATATTAGATCTATTAATGACTAGATAGTTAATCT ATGAGAGTAACTATATTAGATCTATTAATGACTAGATAGTTAATCT
                                  ATGAGAGTAACTATATTAGATCTATTAATGACTAGATAGTTAATCT ATGAGAGTAACTATATTAGATCTATTAATGACTAGATAGTTAATCT
                                  ATGAGAGTAACTATATTAGATCTATTAATGACTAGATAGTTAAT-T
                                  ATGAGAGTAACTATATTAGATCTATTAATGACTAGATAGTTAATCT

A9                                ATGAGAGTAACTATATTAGATCTATTAATGACTAGATAGTTAAT-T ATGAGAGTAACTATATTAGATCTATTAATGACTAGATAGTTAATCT
                                  ATGAGAGTAACTATATTAGATCTATTAATGACTAGATAGTTAAT-T
                                  ATGAGAGTAACTATATTAGATCTATTAATGACTAGATAGTTAATCT

A10                               ATGAGAGTAACTATATTAGATCTATTAATGACTAGATAGTTAAT-T ATGAGAGTAACTATATTAGATCTATTAATGACTAGATAGTTAATCT
                                  ATGAGAGTAACTATATTAGATCTATTAATGACTAGATAGTTAATCT ATGAGAGTAACTATATTAGATCTATTAATGACTAGATAGTTAAT-T
                                  ATGAGAGTAACTATATTAGATCTATTAATGACTAGATAGTTAATCT ATGAGAGTAACTATATTAGATCTATTAATGACTAGATAGTTAATCT
                                  ATGAGAGTAACTATATTAGATCTATTAATGACTAGATAGTTAATCT
                                  ATGAGAGTAACTATATTAGATCTATTAATGACTAGATAGTTAATCT

B3                                ATGAGAGTAACTATATTAGATCTATTAATGACTAGATAGTTAATCT ATGAGAGTAACTATATTAGATCTATTAATGACTAGATAGTTAAT-T
                                  ATGAGAGTAACTATATTAGATCTATTAATGACTAGATAGTTAATCT ATGAGAGTAACTATATTAGATCTATTAATGACTAGATAGTTAAT-T
                                  ATGAGAGTAACTATATTAGATCTATTAATGACTAGATAGTTAAT-T ATGAGAGTAACTATATTAGATCTATTAATGACTAGATAGTTAATCT
                                  ATGAGAGTAACTATATTAGATCTATTAATGACTAGATAGTTAATCT
                                  ATGAGAGTAACTATATTAGATCTATTAATGACTAGATAGTTAATCT
```

B.

```
16/16   TGATGCTCCCTGGAGTTGCCGTTGCTGCGTGCTGAAGGGGATTAATTAAT   ACAAGGTTTCTCACTATCAAGATGTACTGGAGTATAAGAATGAAGAACAG
```

Supplementary Figure s4. Sequences of edited YMR262 loci with G/C poor 46 bp repeats. **(A)** Sequences of repeat regions in isolated edited clones. Clone numbers are indicated on the left. Indels and a deletion are indicated in grey. **(B)** Sequence of the surrounding junctions, 50 bp upstream and downstream of the repeat sequence. Ratio on the left indicates the number of clones of a given sequence over the total number of junctions sequenced.

A.

A1 ATGAGAGTAGCGATGCTAGATCGATGACCGGCTAGATAGTCGATCG ATGAGAGTAGCGATGCTAGATCGATGACCGGCTAGATAGTCGATCG
ATGAGAGTAGCGATGCTAGATCGATGACCGGCTAGATAGTCGATCG ATGAGAGTAGCGATGCTAGATCGATGACCGGCTAGATAGTCGATCG
ATGAGAGTAGCGATGCTAGATCGATGACCGGCTAGATAGTCGATCG

A2 ATGAGAGTAGCGATGCTAGATCGATGACCGGCTAGATAGTCGATCG ATGAGAGTAGCGATGCTAGATCGATGACCGGCTAGATAGTCGATCG
ATGAGAGTAGCGATGCTAGATCGATGACCGGCTAGATAGTCGATCG ATGAGAGTAGCGATGCTAGATCGATGACCGGCTAGATAGTCGATCG
ATGAGAGTAGCGATGCTAGATCGATGACCGGCTAGATAGTCGATCG ATGAGAGTAGCGATGCTAGATCGATGACCGGCTAGATAGTCGATCG

A3,A7,B1,B3,B4,B9,B10 ATGAGAGTAGCGATGCTAGATCGATGACCGGCTAGATAGTCGATCG

A5,A6,A8,A9,B8 ATGAGAGTAGCGATGCTAGATCGATGACCGGCTAGATAGTCGATCG ATGAGAGTAGCGATGCTAGATCGATGACCGGCTAGATAGTCGATCG

A10 ATGAGAGTAGCGATGCTAGATCGATGACCGGCTAGATAGTCGATCG ATGAGAGTAGCGA---------------------------------

B2 ATGAGAGTAGCGATGCTAGATCGATGACCGGCTAGATAGTCGA-CG ATGAGAGTAGCGATGCTAGATCGATGACCGGCTAGATAGTCGATCG

B.

15/16 TGATGCTCCCTGGAGTTGCCGTTGCTGCGTGCTGAAGGGGATTAATTAAT ACAAGGTTTCTCACTATCAAGATGTACTGGAGTATAAGAATGAAGAACAG

A9 TGATGCTCCCTGGAGTTGCCGTTGCTGCGTGCTGAAGGGGATTAATTAAT ACAAGGTTTCTCACTATCAA*C*ATGTACTGGAGTATAAGAATGAAGAACAG

Supplementary Figure s5. Sequences of edited YMR262 loci with G/C neutral 46 bp repeats. **(A)** Sequences of repeat regions in isolated edited clones. Clone numbers are indicated on the left. Indels and a deletion are indicated in grey. (**B**) Sequence of the surrounding junctions, 50 bp upstream and downstream of the repeat sequence. A single nucleotide polymorphism is italicized and underlined. Ratio on the left indicates the number of clones of a given sequence over the total number of junctions sequenced.

A.

A1,A2,A3,A4,A5,A6,A7,A9,A10,B1,B2,B3,B6,B7	ACGAGCGTCGCGAGGCCGGATCGGCGACCGGCTCGCGAGTCGAGCG
A8	ACGAGCGTCGCGAGGCCGGATCGGCGACCGGCTCGCGAGTC*A*AGCG
B8	----------------------GGCGACCGGCTCGCGAGTCGAGCG

B.

15/16	TGATGCTCCCTGGAGTTGCCGTTGCTGCGTGCTGAAGGGGATTAATTAAT	ACAAGGTTTCTCACTATCAAGATGTACTGGAGTATAAGAATGAAGAACAG
B8	TGATGCTCCCTGGAGTTGCCGTTGCTGCGTGCTGAAGGGGATTAATTAATTCCAGG	ACAAGGTTTCTCACTATCAAGATGTACTGGAGTATAAGAATGAAGAACAG

Supplementary Figure s6. Sequences of edited YMR262 loci with G/C rich 46 bp repeats. **(A)** Sequences of repeat regions in isolated edited clones. Clone numbers are indicated on the left. An indel in clone A8 is italicized and underlined. The deletion in clone B8 is indicated in grey. **(B)** Sequence of the surrounding junctions, 50 bp upstream and downstream the repeat sequence. Ratio on the left indicates the number of clones of a given sequence over the total number of junctions sequenced. Insertion in clone B8 at the junction with the repeats is shown in grey.

A.

A5,A6,A8	ATATGATATAAAGTTAGTCTATACTGTATTATTCAATCATAGATTAATTGTTATCATCTATTCTATACATATGACTACTAGTCATAACGTCGTATATTGAGCTGCTATTCAATAATCTAGATCTGATAATAATTAGATTCTTTCACTAGAAAGCTCTAATATATG
	ATATGATATAAAGTTAGTCTATACTGTATTATTCAATCATAGATTAATTGTTATCATCTATTCTATACATATGACTACTAGTCATAACGTCGTATATTGAGCTGCTATTCAATAATCTAGATCTGATAATAATTAGATTCTTTCACTAGAAAGCTCTAATATATG
	ATATGATATAAAGTTAGTCTATACTGTATTATTCAATCATAGATTAATTGTTATCATCTATTCTATACATATGACTACTAGTCATAACGTCGTATATTGAGCTGCTATTCAATAATCTAGATCTGATAATAATTAGATTCTTTCACTAGAAAGCTCTAATATATG
A9,B3,B8	ATATGATATAAAGTTAGTCTATACTGTATTATTCAATCATAGATTAATTGTTATCATCTATTCTATACATATGACTACTAGTCATAACGTCGTATATTGAGCTGCTATTCAATAATCTAGATCTGATAATAATTAGATTCTTTCACTAGAAAGCTCTAATATATG
A10	ATATGATATAAAGTTAGTCTATACTGTATTATTCAATCATAGATTAATTGTTATCATCTATTCTATACATATGACTACTAGTCATAACGTCGTATATTGAGCTGCTATTCAATAA-CT------GATAATAATTAGATTCTTTCACTAGAAAGCTCTAATATATG
	ATATGATATAAAGTTAGTCTATACTGTATTATTCAATCATAGATTAATTGTTATCATCTATTCTATACATATGACTACTAGTCATAACGTCGTATATTGAGCTGCTATTCAATAATCTAGATCTGATAATAATTAGATTCTTTCACTAGAAAGCTCTAATATATG
	ATATGATATAAAGTTAGTCTATACTGTATTATTCAATCATAGATTAATTGTTATCATCTATTCTATACATATGACTACTAGTCATAACGTC*A*TATATTGAGCTGCTATTCAATAAT--AGATCTGATAATAATTAGATTCTTTCACTAGAAAGCTCTAATATATG
	ATATGATATAAAGTTAGTCTATACTGTATTATTCAATCATAGATTAATTGTTATCATCTATTCTATACATATGACTACTAGTCATAACGTCGTATATTGAGCTGCTATTCAATAAT--AGATCTGATAATAATTA*T*ATTCTTTCACTA*T*AAAGCTCT*C*ATATATG
B4,B5	ATATGATATAAAGTTAGTCTATACTGTATTATTCAATCATAGATTAATTGTTATCATCTATTCTATACATATGACTACTAGTCATAACGTCGTATATTGAGCTGCTATTCAATAATCTAGATCTGATAATAATTAGATTCTTTCACTAGAAAGCTCTAATATATG
	ATATGATATAAAGTTAGTCTATACTGTATTATTCAATCATAGATTAATTGTTATCATCTATTCTATACATATGACTACTAGTCATAACGTCGTATATTGAGCTGCTATTCAATAATCTAGATCTGATAATAATTAGATTCTTTCACTAGAAAGCTCTAATATATG
	ATATGATATAAAGTTAGTCTATACTGTATTATTCAATCATAGATTAATTGTTATCATCTATTCTATACATATGACTACTAGTCATAACGTCGTATATTGAGCTGCTATTCAATAATCTAGATCTGATAATAATTAGATTCTTTCACTAGAAAGCTCTAATATATG
	ATATGATATAAAGTTAGTCTATACTGTATTATTCAATCATAGATTAATTGTTATCATCTATTCTATACATATGACTACTAGTCATAACGTCGTATATTGAGCTGCTATTCAATAATCTAGATCTGATAATAATTAGATTCTTTCACTAGAAAGCTCTAATATATG
B6,B10	ATATGATATAAAGTTAGTCTATACTGTATTATTCAATCATAGATTAATTGTTATCATCTATTCTATACATATGACTACTAGTCATAACGTCGTATATTGAGCTGCTATTCAATAATCTAGATCTGATAATAATTAGATTCTTTCACTAGAAAGCTCTAATATATG
	ATATGATATAAAGTTAGTCTATACTGTATTATTCAATCATAGATTAATTGTTATCATCTATTCTATACATATGACTACTAGTCATAACGTCGTATATTGAGCTGCTATTCAATAATCTAGATCTGATAATAATTAGATTCTTTCACTAGAAAGCTCTAATATATG

B.

7/10	TGATGCTCCCTGGAGTTGCCGTTGCTGCGTGCTGAAGGGGATTAATTAAT	ACAAGGTTTCTCACTATCAAGATGTACTGGAGTATAAGAATGAAGAA--CAG
2/10	TGATGCTCCCTGGAGTTGCCGTTGCTGCGTGCTGAAGGGGATTAATTAAT	ACAAGGTTTCTCACTATCAAGATGTACTG-AGTATAAGAATGAAGAA--CAG
1/10	TGATGCTCCCTGGAGTTGCCGTTGCTGCGTGCTGAAGGGGATTAAT*A*TATG	ACAAGGTTTCTCACTATCAAGATGTACTGG*G*AGTATAAGAATGA*A*AAAACAG

Supplementary Figure s7. Sequences of edited YMR262 loci with G/C poor 165 bp repeats. **(A)** Sequences of repeat regions in isolated edited clones. Clone numbers are indicated on the left. Indels are shown in grey. Single nucleotide polymorphisms are underlined and italicized. **(B)** Sequence of the surrounding junctions, 50 bp upstream and downstream of the repeat sequence. Ratio on the left indicates the number of clones of a given sequence over the total number of junctions sequenced. Indels are shown in grey.

A.

A1	ATATGATGTAGAGCGAGTCGATGCTGTGCTAGTCAATCGTAGGCTAGCTGTGATCGTCTAGTCGATACATCTGACTACTAGTCATAACGTCGTACGTTGAGCTGCTGCTCGCTGATCGAGGTCCGATGATGATGCGATGCTCTCGCTAGACAGCTCTGATCGATG
	ATATGATGTAGAGCGAGTCGATGCTGTGCTAGTCAATCGTAGGCTAGCTGTGATCGTCTAGTCGATACATCTGACTACTAGTCATAACGTCGTACGTTGAGCTGCTGCTCGCTGATCGAGGTCCGATGATGATGCGATGCTCTCGCTAGACAGCTCTGATCGATG
	ATATGATGTA*C*AGCGAGTCGATGCTGTGCTAGTCAATCGTAGGCTAGCTGTGATCGTCTAG*G*CGATACATCTGACTACTAGTCATAACGTCATACGTTGAGCTGCTGCTCGCTGATCGAGGTCCGATGATGATGCGATGCTCTCGCTAGACAGCTCTGATCGATG
	ATATGATGTA*T*AGCGAGTC*A*ATGCTGTGCTAGTCAATCGTAGGCTAGCTGTGATCG*A*CTAGTCGATACATCTGACTACTAGTCATAACGTCGTACGTTGAGCTGCTGCTCGCTGATCGAGGTCCGATGATGATGCGATGCTCTCGCTAGACAGCTCTGATCGATG
A2	ATATGATGTAGAGCGAGTCGATGCT*A*TGCTAGTCAATCGTAGGCTAGCTGTGATCGTCTAGTCGATACATCTGACTACTAGTCATAACGTCGTACGTTGAGCTGCTGCTCGCTGATCGAGGTCCGATG*C*TGATGCGATGCTCTCGCTAGACAGCTCTGATCGATG
	ATATGATGTAGAGCGAGTCGATGCTGTGCTAGTCAATCGTAGGCTAGCTGTGATCGTCTAGTCGATACATCTGACTACTAGTCATAACGTCGTACGTTGAGCTGCTGCTCGCTGATCGAGGTCCGATGATGATGCGATGCTCTCGCTAGACAGCTCTGATCGATG
	ATATGATGTAGAGCGAGTCGATGCTGTGCTAGTCAATCGTAGGCTAGCTGTGATCGTCTAGTCGATACATCTGACTACTAGTCATAACGTCGTACGTTGAGCTGCTGCTCGCTGATCGAGGTCCGATGATGATGCGATGCTCTCGCTAGACAGCTCTGATCGATG
	ATATGATGTA*A*AACGA*A*TC*A*ATGCTGTGC*C*AAGCAATCGTAGGCTAGCTGTGATCG*A*CTA*A*TCGATACATCTGACTAC*C*A*A*TCATAACGTCGTACGTTGAGCTGCTGCTCCCTGATCCAGGTCCCATGATGATGCGATGCTCTCGCTAGACAGCTCTGATCGATG
A4	ATATGATGTAGAGCGAGTCGATGCTGTGCTAGTCAATCGTAGGCTAGCTGTGATCGTCTAGTCGATACATCTGACTACTAGTCATAACGTCGTACGTTGAGCTGCTGCTCGCTGATCGAGGTCCGATGATGATGCGATGCTCTCGCTAGACAGCTCTGATCGATG
	ATATGATGTAGAGCGAGTCGATGCTGTGCTAGTCAATCGTAGGCTAGCTGTGATCGTCTAGTCCATACATCTGACTACTAGTCATAACGTCCTACGTTGAGCTGCTGCTCGCTGATCGAGGTCCGATGATGA
A6	ATATGATGTAGAGCGAGTCGATGCTGTGCTAGTCAATCGTAGGCTAGCTGTGATCGTCTAGTCGATACATCTGACTACTAGTCATAACGTCGTACGTTGAGCTGCTGCTCGCTGATCGA--TG
	ATATGATGTAGAGCGAGTCGATGCTGTGCTAGTCAATCGTAGGCTAGCTGTGATCGTCTAGTCGATACATCTGACTACTAGTCATAACGTCGTACGTTGAGCTGCTGCTCGCTGATCGAGGTCCGATGATGATGCGATGCTCTCGCTAGACAGCTCTGATCGATG
A7,B1,B2,B10	ATATGATGTAGAGCGAGTCGATGCTGTGCTAGTCAATCGTAGGCTAGCTGTGATCGTCTAGTCGATACATCTGACTACTAGTCATAACGTCGTACGTTGAGCTGCTGCTCGCTGATCGAGGTCCGATGATGATGCGATGCTCTCGCTAGACAGCTCTGATCGATG
A8	ATATGATGTAGAGCGAGTCGATGCTGTGCTAGTCAATCGTAGGCTAGCTGTGATCGTCTAGTCGATACATCTGACTACTAGTCATAACGTCGTACGTTGAGCTGCTGCTCGCTGATC*A*AGGTCCGATGATGATGCGATGCTCTCGCTAGACAGCTCTGATCGATG
A9,B5	ATATGATGTAGAGCGAGTCGATGCTGTGCTAGTCAATCGTAGGCTAGCTGTGATCGTCTAGTCGATACATCTGACTACTAGTCATAACGTCGTACGTTGAGCTGCTGCTCGCTGATCGAGGTCCGATGATGATGCGATGCTCTCGCTAGACAGCTCTGATCGATG
	ATATGATGTAGAGCGAGTCGATGCTGTGCTAGTCAATCGTAGGCTAGCTGTGATCGTCTAGTCGATACATCTGACTACTAGTCATAACGTCGTACGTTGAGCTGCTGCTCGCTGATCGAGGTCCGATGATGATGCGATGCTCTCGCTAGACAGCTCTGATCGATG
	ATATGATGTAGAGCGAGTCGATGCTGTGCTAGTCAATCGTAGGCTAGCTGTGATCGTCTAGTCGATACATCTGACTACTAGTCATAACGTCGTACGTTGAGCTGCTGCTCGCTGATCGAGGTCCGATGATGATGCGATGCTCTCGCTAGACAGCTCTGATCGATG
	ATATGATGTAGAGCGAGTCGATGCTGTGCTAGTCAATCGTAGGCTAGCTGTGATCGTCTAGTCGATACATCTGACTACTAGTCATAACGTCGTACGTTGAGCTGCTGCTCGCTGATCGAGGTCCGATGATGATGCGATGCTCTCGCTAGACAGCTCTGATCGATG
	ATATGATGT
B6	ATATGATGTAGAGCGAGTCGATGCTGTGCTAGTCAATCGTAGGCTAGCTGTGATCGTCTAGTCGATACATCTGACTACTAGTCATAACGTCGTACGTTGAGCTGCTGCTCGCTGATCGAGGTCCGATGATGATGC-GATGCTCTCGC-TAGACAGCTCTGATCGATG
	ATATGATGTAGAGCGAGTCGATGCTGTGCTAGTCAATCGTAGGCTAGCTGTGATCGTCTAGTCGATACATCTGACTACTAGTCATAACGTCGTACGTTGAGCTGCTGCTCGCTGATCGAGGTCCGATGATGATGC-GATGCTCTCGC-TAGACAGCTCTGATCGATG
	ATATGATGTAGAGCGAGTCGATGCTGTGCTAGTCAATCGTAGGCTAGCTGTGATCGTCTAGTCGATACATCTGACTACTAGTCATAACGTCGTACGTTGAGCTGCTGCTCGCTGATCGAGGTCCGATGATGATGC-GATGCTCTCGC-TAGACAGCTCTGATCGATG
	ATATGATGTAGAGCGAGTCGATGCTGTGCTAGTCAATCGTAGGCTAGCTGTGATCGTCTAGTCGATACATCTGACTACTAGTCATAACGTCGTACGTTGAGCTGCTGCTCGCTGATCGAGGTCCGATGATGATGC-GATGCTCTCGC-TAGACAGCTCTGATCGATG
	ATATGATGTAGAGCGAGTCGATGCTGTGCTAGTCAATCGTAGGCTAGCTGTGATCGTCTAGTCGATACATCTGACTACTAGTCATAACGTCGTACGTTGAGCTGCTGCTCGCTGATCGAGGTCCGATGATGATGCGATGCTCTCGC*T*AGACAGCTCTTGATCGATG*A*
	ATATGATGTAGAGCGAGTCCG
B7	ATATGATGTAGAGCGAGTCGATGCTGTGCTAGTCAATCGTAGGCTAGCTGTGATCGTCTAGTCGATACATCTGACTACTAGTCATAACGTCGTACGTTGAGCTGCTGCTCGCTGATCGAGGTCCGATGATGATGCGATGCTCTCGCTAGACAGCTCTGATCGATG
	ATATGATGTAGAGCGAGTCGATGCTGTGCTAGTCAATCGTAGGCTAGCTGTGATCGTCTAGTCGATACATCTGACTACTAGTCATAACGTCGTACGTTGAGCTGCTGCTCGCTGATCGAGGTCCG-TGATGATGCGATGCTCTCGCTAGACAGCTCTGATCGATG
	ATATGATGTAGAGCGAGTC*A*ATGCTGTGCTAGTCAATCGTAGGCTAGCTGTGATCGTCTAGTCGATACATCTGACTACTAGTCATAACGTC*T*TACGTTGAGCTGCTGCTC*T*CTGATCGAGGTCC
B8	ATATGATGTAGAGCGAGTCGATGCTGTGCTAGTCAATCGTAGGCTAGCTGTGATCGTCTAGTCGATACATCTGACTACTAGTCATAACGTCGTACGTTGAGCTGCTGCTCGCTGATCGAGGTCCGATGATGATGCGATGCTCTCGCTAGACAGCTCTGATCGATG
	ATATGATGTAGAGCGAGTCGATGCTGTGCTAGTCAATCGTAGGCTAGCTGTGATCGTCTAGTCGATACATCTGACTACTAGTCATAACGTCGTACGTTGAGCTGCTGCTCGCTGATCGAGGTCCGATGATGATGCGATGCTCTCGCTAGACAGCTCTGATCGATG
	ATATGATGTAGAGCGAGTCGATGCTGTGCTAGTCAATCGTAGGCTAGCTGTGATCGTCTAGTCGATACATCTGACTACTAGTCATAACGTCGTACGTTGAGCTGCTGCTCGCTGATCGAGGTCCGATGATGATGCGATGCTCTCGCTAGACAGCTCTGATCGATG
B9	ATATGATGTAGAGCGAGTCGATGCTGTGCTAGTCAATCGTAGGCTAGCTGTGATCGTCTAGTCGATACATCTGACTACTAGTCATAACGTCGTACGTTGAGCTGCTGCTCGCTGATCGAGGTCCGATGATGATGCGATGCTCTCGCTAGACAGCTCTGATCGATG
	ATATGATGTAGAGCGAGTCGATGCTGTGCTAGTCAATCGTAGGCTAGCTGTGATCGTCTAGTCGATACATCTGACTACTAGTCATAACGTCGTACGTTGAGCTGCTGCTCGCTGATCGAGGTCCGATGATGATGCGATGCTCTCGCTAGACAGCTCTGATCGATG
	ATATGATGTAGAGCGAGTCGATGCTGTGCTAGTCAATCGTAGGCTAGCTGTGATCGTCTAGTCGATACATCTGACTACTAGTCATAACGTCGTACGTTGAGCTGCTGCTCGCTGATCGAGGTCCGATGATGATGCGATGCTCTCGCTAGACAGCTCTGATCGATG
	ATATGATGTAGAGCGAGTCGATGCTGTGCTAGTCAATCGTAGGCTAGCTGTGATCGTCTAGTCGATACATCTGACTACTAGTCATAACGTCGTACGTTGAGCTGCTGCTCGCTGATCGAGGTCCGATGATGATGCGATGCTCTCGCTAGACAGCTCTGATCGATG
	ATATGATGTAGAGCGAGTCGATGCTGTGCTAGTCAATCGTAGGCTAGCTGTGATCGTCTAGTCGATACATCTGACTACTAGTCATAACGTCGTACGTTGAGCTGCTGCTCGCTGATCGAGGTCCGATGATGATGCGATGCTCTCGCTAGACAGCTCTGATCGATG
	ATATGATGTAGAGCGAGTCGATGCTGTGCTAGTCAATCGTAGGCTAGCTGTGATCGTCTAGTCGATACATCTGACTACTAGTCATAACGTCGTACGTTGAGCTGCTGCTCGCTGATC*A*AGGTCCGATGATGATGCGATGCTCTCGCTAGACAGCTCTGATCGATG
	ATAT

B.

11/11	TGATGCTCCCTGGAGTTGCCGTTGCTGCGTGCTGAAGGGGATTAATTAAT	ACAAGGTTTCTCACTATCAAGATGTACTGGAGTATAAGAATGAAGAACAG

Supplementary Figure s8. Sequences of edited YMR262 loci with G/C neutral 165 bp repeats. **(A)** Sequences of repeat regions in isolated edited clones. Clone numbers are indicated on the left. A large deletion in clone A6 is indicated in grey. Single nucleotide polymorphisms are italicized

and underlined. **(B)** Sequence of the surrounding junctions, 50 bp upstream and downstream the repeat sequence. Ratio on the left indicates the number of clones of a given sequence over the total number of junctions sequenced.

A.

```
A1,A2,A5,A8,B2,B4  AGATGCCGCGGCGCGAGTCGACGCTGTGCTGGCCGGTCGTCGGCTCGCTGGGATCGTCTCGGCGATACGTCGGGCTACTCGGCCTCACGTCGCGCGTCGCGCTGCGGCTCGCCGAGCGCGGTCCGAGGGCGATGCGCTGCTCTCGCGCGCCGGCTCGGATCGCTG

A3,A6              AGATGCCGCGGCGCGAGTCGACGCTGTGCTGGCCGGTCGTCGGCTCGCTGGGATCGTCTCGGCGATACGTCGGGCTACTCGGCCTCACGTCGCGCG-----------------------------------------------------CCGGCTCGGATCGCTG

A4                 AGATGCCGCGGCGCGAGTCGACGCTGTGCTGGCCGGTCGTCGGCTCGCTGGGATCGTCTCGGCGATACGTCGGGCTACTCGGCCTCACGTCGCGCGTCGCGCTGC-----------------------------------TCTCGCGCGCCGGCTCGGATCGCTG

A7                 AGATGCCGCGGCGCGAGTCGACGCTGTGCTGGCCGGTCGTCGGCTCGCTGGGATCGTCTCGGCGATACGTCGGGCTACTCGGCCTCACGTCGCGCGTCGCGCTGCGGCTCGCCGAGCGCGGTCCGAGGGCGATGCGCTGCTCTCGCGCGCCGGCTCGGATCGCTG
                   AGATGCCGCGGCGCGAGTCGACGCTGTGCTGGCCGGTCGTCGGCTCGCTGGGATCGTCTCGGCGATACGTCGGGCTACTCG-CCTCACGTCGCGCGTCGCGCTGCGGCTCGCCGAGCGCGGTCCGAGGGCGATGCGCTGCTCTCGCGCGCCGGCTCGGATCGCTG

A9                 AGATGCCGCGGCGCGAGTCGACGCTGTGCTGGCCGGTCGTCGGCTCGCTG-------------------------------------------------------------------------------------------------------------------
                   AGATGCCGCGGCGCGAGTCGACGCTGTGCTGGCCGGTCGTCGGCTCGCTGGGATCGTCTCGGCGATACGTCGGGCTACTCGGCCTCACGTCGCGCGTCGCGCTGCGGCTCGCCGACGGGTG-----GGGCGATGCGCTGCTCTCGCGCGCCGGCTCGGATCGCTG

B1                 AGATGCCGCGGCGCGAGTCGACGCTGTGCTGGCCGGTCGTCGGCTCGCTGGGATCGTCTCGGCGATACGTCGGGCTACTCGGCCTCACGTCGCGCGTCGCGCTGCGACTCGCC-AGCGCGGTCCGAGGGCGATGCGCTGCTCTCGCGCGCCGGCTCGGATCGCTG

B3                 AGATGCCGCGGCGCGAGTCGACGCTGTGCTGGGCCGGTCGTCGGCTCGCTGGGATCGTCT-GGCGATACGTCGGGCTACTCGGCCTCACGTCGCGCG----------------------------------------------------CCGGCTCGGATCGCTG

B6                 AGATGCCGCGGCGCGAGTCGACGCTGTGCTGGCCGGTCGTCGGCTCGCTGGGATCGTCTCGGCGATACGTCGGGCTACTCGGCCTCACGTCGCGCGTCGCG----------------------------------------------------------------
                   ---------------AGTCGACGCTGTGCTGGCCGGTCGTCGGCTCGCTGGGATCGTCTCGGCGATACGTCGGGCTACTCGGCCTCACGTCGCGCGTCGCGCTGCGGCTCGCCGAGCGCGGTCCGAGGGCGATGCGCTGCTCTCGCGCGCCGGCTCGGATCGCTG

B9                 AGATGCCGCGGCGCGAGTCGACGCTGTGCTGGCCGGTCGTCGGCTCGCTGGGATCGTCTCGGCGATACGTCGGGCTACTCGGCCTCACGTCGCGCGTCGCGCTGCG-CTCGCCGAGCGCGGTCCGAGGGCGATGCGCTGCTCTCGCGCGCCGGCTCGGATCGCTG

B10                AGATGCCGCGGCGCGAGTCGACGCTGTGCTGGCCGGTCGTCGGCTCGCTGGGATCGTCTCGGCGATACGTCGGGCTACTCGGCCTCACGTCGCGCGTCGCGCTGCGGCTCGCCGAGCGCGGTCCGAGGGCGATGCGCTGCTCTCGCGCGCCGGCTCGGATCGCTG
                   AGATGCCGCGGCGCGAGTCGACGCTGTGCTGGCCGGTCGTCGGCTCGCTGGGATCGTCTCGGCGATACGTCGGGCTACTCGGCCTCACGTCGCGCGTCGCGCTGCGGCTCGCCGAGCGCGGTCCGAGGGCGATGCGCTGCTCTCGCGCGCCGGCTCGGATCGCTG
```

B.

```
15/15     TGATGCTCCCTGGAGTTGCCGTTGCTGCGTGCTGAAGGGGATTAATTAAT     ACAAGGTTTCTCACTATCAAGATGTACTGGAGTATAAGAATGAAGAACAG
```

Supplementary Figure s9. Sequences of edited YMR262 loci with G/C rich 165 bp repeats. **(A)** Sequences of repeat regions in isolated edited clones. Clone numbers are indicated on the left. Indels and large deletions are indicated in grey. Single nucleotide polymorphisms are italicized and underlined **(B)** Sequence of the surrounding junctions, 50 bp upstream and downstream of the repeat sequence. Ratio on the left indicates the number of clones of a given sequence over the total number of junctions sequenced.

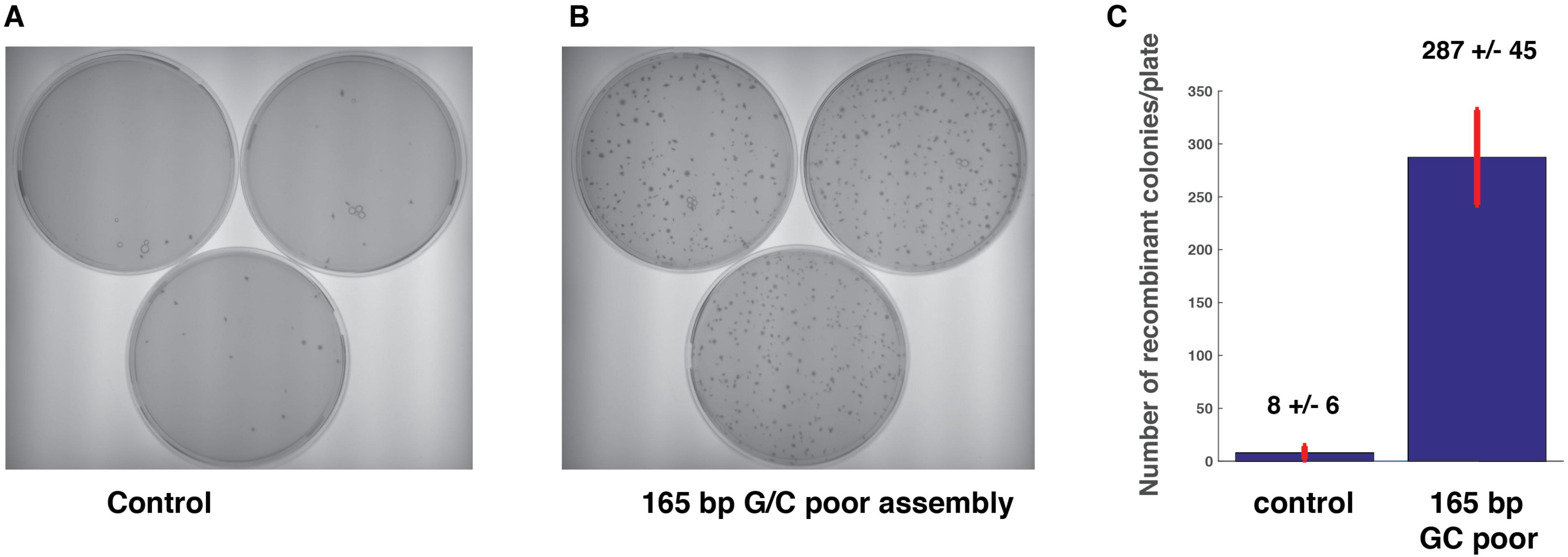

Supplementary Figure s10. Expected results obtained after transformation of the Cas9-expressing strain with **(A)** the plasmid expressing the guide RNA targeting the YMR262 gene (control) or **(B)** the guide RNA expressing plasmid + donor PCRs + repeat oligonucleotides (165 bp GC poor oligos in this case, see Material and Methods and supplementary Table s3 for details). Results obtained from 3 independent transformations for each condition. **(C)** Quantification of the number of colonies/plate from the three independent transformations shown in A and B. mean +/- standard deviation is displayed.

Supplementary information

Supplementary Table s1. Details of engineered repeats

**Design name (number of oligonucleotides used)**	**Sequence of the expected monomer**
G/C poor 4 bp (2)	ATGA
G/C neutral 4 bp (1)	ATGC
G/C rich 4 bp (2)	ACGC
G/C poor 46 bp (2)	ATGAGAGTAACTATATTAGATCTATTAATGACTAGATAGTTAATCT
G/C neutral 46 bp (2)	ATGAGAGTAGCGATGCTAGATCGATGACCGGCTAGATAGTCGATCG
G/C rich 46 bp (2)	ACGAGCGTCGCGAGGCCGGATCGGCGACCGGCTCGCGAGTCGAGCG
G/C poor 165 bp (4)	ATATGATATAAAGTTAGTCTATACTGTATTATTCAATCATAGATTAA<br>TTGTTATCATCTATTCTATACATATGACTACTAGTCATAACGTCGTA<br>TATTGAGCTGCTATTCAATAATCTAGATCTGATAATAATTAGATTCT<br>TTCACTAGAAAGCTCTAATATATG
G/C neutral 165 bp (4)	ATATGATGTAGAGCGAGTCGATGCTGTGCTAGTCAATCGTAGGCTAG<br>CTGTGATCGTCTAGTCGATACATCTGACTACTAGTCATAACGTCGTA<br>CGTTGAGCTGCTGCTCGCTGATCGAGGTCCGATGATGATGCGATGCT<br>CTCGCTAGACAGCTCTGATCGATG
G/C rich 165 bp (4)	AGATGCCGCGGCGCGAGTCGACGCTGTGCTGGCCGGTCGTCGGCTCG<br>CTGGGATCGTCTCGGCGATACGTCGGGCTACTCGGCCTCACGTCGCG<br>CGTCGCGCTGCGGCTCGCCGAGCGCGGTCCGAGGGCGATGCGCTGCT<br>CTCGCGCGCCGGCTCGGATCGCTG

Supplementary Table s2. **Strains used in this study**

Strain name	Genotype	Reference
**YPH499**	MATa *ura3-52 lys2-801_amber ade2-101_ochre trp1-Δ63 his3-Δ200 leu2-Δ1*	ATCC # 204679
**ALY0**	YPH499 + pAL30	This study
**ALY1.1-20**	ALY0 *ymr262::165-Rich*	This study
**ALY2.1-20**	ALY0 *ymr262::165-Neutral*	This study
**ALY3.1-20**	ALY0 *ymr262::165-Poor*	This study
**ALY4.1-20**	ALY0 *ymr262::46-Rich*	This study
**ALY5.1-20**	ALY0 *ymr262::46-Neutral*	This study
**ALY6.1-20**	ALY0 *ymr262::46-Poor*	This study
**ALY7.1-20**	ALY0 *ymr262::4-Rich*	This study
**ALY8.1-20**	ALY0 *ymr262::4-Neutral*	This study
**ALY9.1-20**	ALY0 *ymr262::4-Poor*	This study

Supplementary Table s3. **Plasmids used in this study**

Plasmid Name	Description	Reference	plasmids/oligos
**p414-Cas9**	p414-TEF1p-Cas9-CYC1t	Addgene #43802	-
**pRS413**	pRS413	[1]	-
**pAL30**	pRS413-Cas9	This study	pRS413 ; Addgene #43802
**p426**	p426-SNR52p-gRNA.CAN1.Y-SUP34t	Addgene #43803	-
**pAL31**	p426-SNR52p-gRNA.YMR262-SUP34t	This study	Addgene #43803 / AL-O-44-47

__Supplementary Table s4.__ **List of primers used in this study. Sequences homologous to genomic DNA in oligonucleotides AL-O-25 to AL-O-42 are highlighted in bold. The guide RNA sequence targeting YMR262 is shown in italics and underlined.**

Primer Name	Sequence	Description
**AL-O-01**	AGATGCCGCGGCGCGAGTCGACGCTGTGCTGGCCGGTCGTCGGCTCGCTGGGATCGTCTC	165-Rich-f1
**AL-O-02**	CGCGACGCGCGACGTGAGGCCGAGTAGCCCGACGTATCGCCGAGACGATCCCAGCGAGCC	165-Rich-r2
**AL-O-03**	CCTCACGTCGCGCGTCGCGCTGCGGCTCGCCGAGCGCGGTCCGAGGGCGATGCGCTGCTC	165-Rich-f3
**AL-O-04**	ACTCGCGCCGCGGCATCTCAGCGATCCGAGCCGGCGCGCGAGAGCAGCGCATCGCCCTCG	165-Rich-r4
**AL-O-05**	ATATGATGTAGAGCGAGTCGATGCTGTGCTAGTCAATCGTAGGCTAGCTGTGATCGTCTA	165-Neutral-f1
**AL-O-06**	CTCAACGTACGACGTTATGACTAGTAGTCAGATGTATCGACTAGACGATCACAGCTAGCC	165-Neutral-r2
**AL-O-07**	CATAACGTCGTACGTTGAGCTGCTGCTCGCTGATCGAGGTCCGATGATGATGCGATGCTC	165-Neutral-f3
**AL-O-08**	ACTCGCTCTACATCATATCATCGATCAGAGCTGTCTAGCGAGAGCATCGCATCATCATCG	165-Neutral-r4
**AL-O-09**	ATATGATATAAAGTTAGTCTATACTGTATTATTCAATCATAGATTAATTGTTATCATCTA	165-Poor-f1
**AL-O-10**	CTCAATATACGACGTTATGACTAGTAGTCATATGTATAGAATAGATGATAACAATTAATC	165-Poor-r2
**AL-O-11**	CATAACGTCGTATATTGAGCTGCTATTCAATAATCTAGATCTGATAATAATTAGATTCTT	165-Poor-f3
**AL-O-12**	ACTAACTTTATATCATATCATATATTAGAGCTTTCTAGTGAAAGAATCTAATTATTATCA	165-Poor-r4
**AL-O-13**	ACGAGCGTCGCGAGGCCGGATCGGCGACCGGCTCGCG	46-Rich-f1
**AL-O-14**	CCGGCCTCGCGACGCTCGTCGCTCGACTCGCGAGCCGGTCGCCGAT	46-Rich-r2
**AL-O-15**	ATGAGAGTAGCGATGCTAGATCGATGACCGGCTAGAT	46-Neutral-f1
**AL-O-16**	CTAGCATCGCTACTCTCATCGATCGACTATCTAGCCGGTCATCGAT	46-Neutral-r2
**AL-O-17**	ATGAGAGTAACTATATTAGATCTATTAATGACTAGAT	46-Poor-f1
**AL-O-18**	CTAATATAGTTACTCTCATAGATTAACTATCTAGTCATTAATAGAT	46-Poor-r2
**AL-O-19**	ACGCACGCACGCACGCACGCACGCACGCACGCACGCACGC	4-Rich-f1
**AL-O-20**	GCGTGCGTGCGTGCGTGCGTGCGTGCGTGCGTGCGTGCGT	4-Rich-r2

**AL-O-21**	ATGCATGCATGCATGCATGCATGCATGCATGCATGCATGC	4-Neutral
**AL-O-22**	AATGAATGAATGAATGAATGAATGAATGAATGAATGAATG	4-Poor-f1
**AL-O-23**	CATTCATTCATTCATTCATTCATTCATTCATTCATTCATT	4-Poor-r2
**AL-O-24**	AAGCGACGATAATAGTCATTGAGGTTG	forward-left-junction-YMR262/repeats
**AL-O-25**	CGGCCAGCACAGCGTCGACTCGCGCCGCGGCATCT**ATTAATTAATCCCCTTCAGCACGCAGC**	reverse-left-junction-YMR262/165-Rich
**AL-O-26**	TGACTAGCACAGCATCGACTCGCTCTACATCATAT**ATTAATTAATCCCCTTCAGCACGCAGC**	reverse-left-junction-YMR262/165-Neutral
**AL-O-27**	TGAATAATACAGTATAGACTAACTTTATATCATAT**ATTAATTAATCCCCTTCAGCACGCAGC**	reverse-left-junction-YMR262/165-Poor
**AL-O-28**	CGAGCCGGTCGCCGATCCGGCCTCGCGACGCTCGT**ATTAATTAATCCCCTTCAGCACGCAGC**	reverse-left-junction-YMR262/46-Rich
**AL-O-29**	CTAGCCGGTCATCGATCTAGCATCGCTACTCTC**ATTATTAATTAATCCCCTTCAGCACGCAG**	reverse-left-junction-YMR262/46-Neutral
**AL-O-30**	CTAGTCATTAATAGATCTAATATAGTTACTCTCAT**ATTAATTAATCCCCTTCAGCACGCAGC**	reverse-left-junction-YMR262/46-Poor
**AL-O-31**	CGTGCGTGCGTGCGTGCGTGCGTGCGTGCGTGCGTGCGT**ATTAATTAATCCCCTTCAGCACGCAGC**	reverse-left-junction-YMR262/4-Rich
**AL-O-32**	CATGCATGCATGCATGCATGCATGCATGCATGC**ATTATTAATTAATCCCCTTCAGCACGCAGC**	reverse-left-junction-YMR262/4-Neutral
**AL-O-33**	CATTCATTCATTCATTCATTCATTCATTCATTC**ATTATTAATTAATCCCCTTCAGCACGCAGC**	reverse-left-junction-YMR262/4-Poor
**AL-O-34**	GATGCGCTGCTCTCGCGCGCCGGCTCGGATCGCTG**ACAAGGTTTCTCACTATCAAGATGTACTGG**	forward-right-junction-YMR262/165-Rich
**AL-O-35**	GATGCGATGCTCTCGCTAGACAGCTCTGATCGATG**ACAAGGTTTCTCACTATCAAGATGTACTGG**	forward-right-junction-YMR262/165-Neutral
**AL-O-36**	AATTAGATTCTTTCACTAGAAAGCTCTAATATATG**ACAAGGTTTCTCACTATCAAGATGTACTGG**	forward-right-junction-YMR262/165-Poor
**AL-O-37**	GAGGCCGGATCGGCGACCGGCTCGCGAGTCGAGCG**ACAAGGTTTCTCACTATCAAGATGTACTGG**	forward-right-junction-YMR262/46-Rich
**AL-O-38**	GATGCTAGATCGATGACCGGCTAGATAGTCGATCGG**ACAAGGTTTCTCACTATCAAGATGTACTGG**	forward-right-junction-YMR262/46-Neutral
**AL-O-39**	ATATTAGATCTATTAATGACTAGATAGTTAATCT**ACAAGGTTTCTCACTATCAAGATGTACTGG**	forward-right-junction-YMR262/46-Poor
**AL-O-40**	CGCACGCACGCACGCACGCACGCACGCACGCACGC**ACAAGGTTTCTCACTATCAAGATGTACTGG**	forward-right-junction-YMR262/4-Rich

**AL-O-41**	TGCATGCATGCATGCATGCATGCATGCATGCATGC**ACAAGGTTTCTCACTATCAAGATG TACTGG**	forward-right-junction-YMR262/4-Neutral
**AL-O-42**	AATGAATGAATGAATGAATGAATGAATGAATGAATG**ACAAGGTTTCTCACTATCAAGAT GTACTGG**	forward-right-junction-YMR262/4-Poor
**AL-O-43**	TACTCTTCAAGATCTAGAGGTTCGGGAAG	reverse-right-junction-YMR262/repeats
**AL-O-44**	CGGCCGCGTATCGATCATTTATCTTTCACTGC	p426- YMR262-ClaI
**AL-O-45**	CGGCCGCCGGTACCCAATTCGCCCTATAG	p426- YMR262-KpnI
**AL-O-46**	CGGCCGATCGAT*TGTGGGAAGTCGGCGCGACA*GTTTTAGAGCTAGAAATAGCAAGT	YMR262-gRNA cassette-forward
**AL-O-47**	GGCGAATTGGGTACCGGCCGC	YMR262- gRNA cassette-reverse
**AL-O-48**	GCCAGGACCTGCAATAACGTTTGTAC	forward-repeats-sequencing
**AL-O-49**	TACTCTTCAAGATCTAGAGGTTCGGGAAG	reverse-repeats-sequencing
**AL-O-50**	GAGCAGCTGCAGTATTTGAATGCAC	forward-southern-genomic-probe
**AL-O-51**	GCTACCCGCACCGTATACAAAAAGG	reverse-southern-genomic-probe

Supplementary references

1. Sikorski, R. S. & Hieter, P. A system of shuttle vectors and yeast host strains designed for efficient manipulation of DNA in Saccharomyces cerevisiae. *Genetics* **122,** 19–27 (1989).
